# A functional mutation associated with piglet diarrhea partially by regulating the transcription of porcine *STAT3*

**DOI:** 10.3389/fvets.2022.1034187

**Published:** 2022-11-04

**Authors:** Zhihua Chen, Diwen Yao, Dongchun Guo, Yuan Sun, Lu Liu, Mingxing Kou, Xiuqin Yang, Shengwei Di, Jiancheng Cai, Xibiao Wang, Buyue Niu

**Affiliations:** ^1^College of Animal Science and Technology, Northeast Agricultural University, Harbin, China; ^2^State Key Laboratory of Veterinary Biotechnology, Harbin Veterinary Research Institute, Chinese Academy of Agricultural Sciences (CAAS), Harbin, China; ^3^Lanxi Breeding Farm, Lanxi, China

**Keywords:** porcine, STAT3, polymorphism, promoter, piglet diarrhea

## Abstract

The present study aimed to search for functional mutations within the promoter of porcine *STAT3* and to provide causative genetic variants associated with piglet diarrhea. We firstly confirmed that *STAT3* expressed higher in the small intestine than in the spleen, stomach and large intestine of SPF piglets, respectively (*P* < 0.05). Then, 10 genetic variations in the porcine STAT3 promoter region was identified by direct sequencing. Among them, three mutations SNP1: g.−870 G>A, SNP2: g.−584 A>C and a 6-bp Indel in the promoter region that displayed significant differential transcriptional activities were identified. Association analyses showed that SNP1: g.−870 G>A was significantly associated with piglet diarrhea (*P* < 0.05) and the GG animals had lower diarrhea score than AA piglets (*P* < 0.01) in both Min and Landrace population. Further functional analysis revealed that E2F6 repressed the transcriptional efficiency of *STAT3 in vitro*, by binding the G allele of SNP1. The present study suggested that SNP1: g.−870 G>A was a piglet diarrhea-associated variant that directly affected binding with E2F6, leading to changes in *STAT3* transcription which might partially contribute to piglet diarrhea susceptibility or resistance.

## Introduction

Pork produced by the modern pig industry accounts for more than one third of global meat consumption (1). Piglet diarrhea, caused by multiple factors including pathogen infection, nutrition and management, is a major threat to pig production. Traditionally, the focus of control efforts against animal disease have been on vaccination, veterinary drugs, feed additive and biosecurity (2), but drug residues and antibiotic resistance are inevitable hazards to animal health, pork security and the external environment. An alternative method is now focused on breeding animals that genetically respond more robustly to infection, improving their health and welfare and ultimately resulting in safe and high-quality pork.

Genetic-based selective breeding firstly involves identifying causative genetic variants associated with animal health. Traditional studies focused on the protein-coding regions to identify trait-associated variants which altered the amino acid sequences and eventual protein functions. For example, Holder et al. (3) reported a single nucleotide polymorphism (SNP) c.1066C>G in the bovine *SLC11A1* gene associated with diverse expression of *NRAMP1* in bovine macrophages. It is now widely accepted that the promoter region is critical for identifying variants associated with complex traits (4, 5). Recent research demonstrated a close link between mutations within transcription factor binding sites (TFBSs) and gene transcription or expression. Niu et al. (6) showed that the CEBPB binding polymorphism in the porcine *CXCL14* promoter contributed to the differential expression of *CXCL14* mRNA in two pig breeds with diverse resistance to porcine reproductive and respiratory syndrome virus (PRRSV). Liu et al. (7) showed that a 14-bp insertion in the promoter region increased porcine *MRC1* transcription after porcine circovirus type 2 (PCV2) infection. But literature on the identification of causative SNPs associated with piglet diarrhea is limited.

Signal transducers and activators of transcription (STAT) are a family of ubiquitous transcription factors located at the junction of several cytokine-signaling pathways which exercises multiple cellular functions. Like other STAT family members, STAT3 is known to exert widespread effects in immunity and disease through mediating a variety of specific gene transcription responses to cytokines (8–10). Human STAT3 plays a significant role in the pathogenesis of herpesviruses and varicella-zoster virus (VZV) as shown by Sen et al. and *STAT3* mutations cause autosomal dominant hyper-IgE syndrome, a rare disorder of immunity characterized by elevated IgE levels and recurrent bacterial infections in skin and lung (11–15). Genetic variants in the *STAT3* has been found to alter individual susceptibility to inflammatory bowel disease (16, 17), a complex gastrointestinal disease characterized by chronic relapsing intestinal inflammation.

Porcine epidemic diarrhea virus (PEDV) is closely linked to piglet viral diarrhea (18, 19). Yang et al. (20) showed that PEDV infection actived epidermal growth factor receptor (EGFR) and the downstream STAT3, both impaired the antiviral activity of type I interferon and facilitated PEDV replication in the porcine epithelial cell line J2 (IPEC-J2). Hu et al. (21) found STATs, including *STAT1, STAT2, STAT3, STAT4*, and *STAT5A*, were significantly induced by PEDV infection in IPEC-J2. Subsequently, the bioinformatics analysis revealed that 59.02% of the differentially expressed genes after PEDV infection possess binding sites for STATs in the putative promoter regions. Piglet weaning stress is another common factor for piglet diarrhea. Yi et al. (22) found weaning caused intestinal inflammation and activated the STAT3 signaling pathways in the jejunum of piglets. Given the vital role of STAT3 in host immunity regulation and the response to PEDV infection, *STAT3* could be regarded as a candidate gene for piglet diarrhea.

It has been reported that pigs vary in their ability to resist diarrhea disease, suggesting the important role of host genetics in disease prevention (23, 24). Indeed, many indigenous pig breeds have been observed to have a lower incidence of diseases compared to general breeds (6, 25). The Min pig is an excellent indigenous breed living in northern China, which has superior meat quality, high reproductive performance and strong resistance to disease (http://afs.okstate.edu/breeds/swine/minzhu/index.html/). Michaels et al. (26) found the frequency of gene which conferring susceptibility to *E. coli* K88 mediated disease was low in Min pig, and Zhang et al. (27) found Min possessed predominant alleles to resist piglet diarrhea. Landrace is a global general breed known to have higher litter size, rapid growth rate, but lower resistance to disease. By observing the severity and recurrence rate of diarrhea from Min and Landrace suckling piglets, our previous study confirmed the Min had a greater resistance to piglet diarrhea (25).

The purpose of this research was to identify functional genetic variations associated with piglet diarrhea. In the current study, porcine *STAT3* was selected as functional candidate gene; then, the genetic variations in porcine *STAT3* promoter were identified and association analysis between polymorphisms and piglet diarrhea score were performed in Min and Landrace populations; lastly, the biological function of specific genetic variation was explored. The outcomes provide novel causative genetic variants for selective breeding to improve piglet health and food safety.

## Materials and methods

### Animal and tissue collection

Min and Landrace piglets from Lanxi Farm (Lanxi, Heilongjiang, China) were observed to obtain the diarrhea score. From birth to weaning at day 35, all the piglets' feces were observed at 6:00 am and 2:00 pm every day, then the daily diarrhea score was assigned to every piglets according to the following standard: normal solid feces 0, slight diarrhea with soft and loose feces 1, moderate diarrhea with semi-liquid feces 2 and severe diarrhea with liquid and unformed feces 3 (28). Piglet diarrhea score was calculated as the summation of the daily diarrhea score during the experiment. All the genomic DNA were extracted from the ear tissues of these piglets and stored at −20°C. In this study, according to the piglet diarrhea score, 20 piglets DNA (5 healthy Min and 5 healthy Landrace piglets with normal solid feces records, 5 diarrhea Min and 5 diarrhea Landrace with high diarrhea score) were used to screen the genetic variation in the porcine STAT3 promoter. A total of 226 Min and 186 Landrace piglets born in the same period from 35 Min sows and 26 Landrace sows were used to perform the association analysis.

For mRNA expression profile analysis, four 28-day SPF piglets provided by Harbin Veterinary Research Institute, Chinese Academy of Agricultural Sciences (CAAS) (Harbin, Heilongjiang, China) were slaughtered and the tissues including spleen, stomach, duodenum, jejunum, ileum and cecum were collected.

### Primer design, polymerase chain reaction and quantitative reverse transcription PCR(RT-qPCR)

Primer pairs used in this study were designed with Primer 5 software using the porcine *STAT3* (NM_001044580.1), *E2F4* (XM_003126933.6), *E2F6* (XM_005655271) or genomic sequences retrieved from Ensembl at http://www.ensembl.org/ in Supplementary Table S1. The PCR reaction contained 100 ng DNA templates, 0.5 μM of each primer as in Supplementary Table S1 and 10 μL 2 × Taq Master Mix (TaKaRa, Dalian, China). The PCR conditions were 4 min at 94°C; 35 cycles of 30 s at 94°C, 30 s at the annealing temperature as in Supplementary Table S1, 1 min at 72°C and a final extension step at 72°C for 10 min.

For the RT-qPCR, total RNA was firstly extracted from piglets tissues or IPEC-J2 cells using TRIzol (Takara, Dalian, China) in line with the manufacturer's protocol and reverse transcribed into cDNA using PrimerScript RT Master Mix (Takara, Dalian, China). According to the protocol of SYBR Premix Ex Taq (Takara, Dalian, China), the qPCR reaction contained 100 ng cDNA, 0.2 μM of each primer as in Supplementary Table S1 and 10 μl SYBR mix (Takara, Dalian, China). The qPCR was conducted on an ABI 7,500 system (Applied Biosystems, Foster city, CA, USA) using the conditions 95°C for 30 s, 40 cycles with 95°C for 5 s and 60°C for 35 s, with the melting curves constructed at the same time. Glyceraldehyde-3-phosphate dehydrogenase (*GAPDH*) was used as reference gene and the relative mRNA expression of a specific gene in different tissues or cells was calculated using the 2^−ΔΔCT^ method (29).

### Genetic variations analysis in porcine *STAT3* promoter region

Porcine *STAT3* promoter region were amplified using the primer pair STAT3-P in Supplementary Table S1 and genomic DNA from 20 animals consisted of five healthy Min, five diarrheal Min, five healthy Landrace and five diarrheal Landrace pigs which were described above. All the PCR products were purified, sequenced commercially (Sangon, Shanghai, China) and assembled for variants analysis with Clustal Omega. Transcription factor binding sites were then predicted using the PROMO (http://alggen.lsi.upc.es/cgi-bin/promo_v3/promo/promoinit.cgi?dirDB=TF_8.3) and JASPAR (http://jaspar.genereg.net/) with a threshold score of 90. The CpG islands were predicted by MethPrimer (http://www.urogene.org/cgi-bin/methprimer/methprimer.cgi) with an expected ratio exceeding 0.6 and G+C exceeding 50%.

### Site-directed mutagenesis and dual luciferase reporter assays

To verify functional genetic variations predicted by JASPAR and PROMO, the *STAT3* promoter region was amplified using the primer pair pGL3-P in Supplementary Table S1 and inserted between the *Kpn* I and *Mlu* I restriction sites of the pGL3-Basic luciferase reporter vector (Promega, Madison, WI, USA), which was named pGL3-STAT3-P. With pGL3-STAT3-P plasmid as the template, three mutants termed SNP1 or g.−870 G>A, SNP2 or g.−584 A>C and SNP3 or g.34 G>C, were generated with mutagenic primers in Supplementary Table S1. Similarly, these fragments were cloned between the *Kpn* I and *Mlu* I sites of the pGL3-Basic vector and named pGL3-STAT3-SNP1, pGL3-STAT3-SNP2, and pGL3-STAT3-SNP3, respectively. In addition, fragments containing 6-bp inserts or deletions (Indel) were amplified with the primer pair listed in Supplementary Table S1 and corresponding genotyped genomic DNA as the template, then inserted into pGL3-Basic vector using *Kpn* I and *Mlu* I restriction sites to construct pGL3-STAT3-In and pGL3-STAT3-Del, respectively. All the positive plasmids were confirmed by DNA sequencing.

Dual luciferase reporter assays were conducted in IPEC-J2 cells with the plasmids constructed above, the pGL3-Basic and pRL-TK (Promega, Madison, Wisconsin, USA). In brief, IPEC-J2 cells were cultured in DMEM supplemented with 10% FBS (Gibco, Carlsbad, CA, USA). After 18 to 24 h, 0.5 μg of reconstructed luciferase reporter plasmids or pGL3-basic as a negative control and 0.005 μg of internal control (pRL-TK) were transiently transfected into cells using 1.5 μl of X-treme GENE HP DNA Transfection Reagent (Roche, USA). After 24 to 48 h, all the cells were lysed and the enzymatic activity of firefly and Renilla were examined using the Dual-Luciferase Reporter Assay System (Promega). Relative luciferase activity was calculated as the ratio of firefly to Renilla. Three replicas were used in cell transfection and the measurement of luciferase.

### Genotyping of the putative functional genetic variations

An enforced *Msp* I PCR-fragment length polymorphism (PCR-RFLP) method was used to genotype SNP1 of porcine *STAT3*. Briefly, a mismatched primer as a G to C mutation at the second base of the 3′ of the forward primer was designed to introduce a recognition site of the restriction enzyme *Msp* I for genotyping SNP1 as in Supplementary Table S1. After PCR amplification, the PCR products were digested with five units of *Msp* I (Takara, Dalian, China) at 37°C for 5 h, separated on an 8% polyacrylamide gel electrophoresis (PAGE).

The SNP2 of the porcine *STAT3* was genotyped by PCR-based single strand conformation polymorphism (PCR-SSCP). In brief, fragments containing SNP2 were amplified by PCR using primer pair in Supplementary Table S1. Then 1 μl PCR products mixed with 9 μl denaturation buffer were denatured for 10 min at 98°C, placed on the iced water for 5 min, separated on a 14% PAGE gel and resolved by silver staining. For the 6-bp Indel of *STAT3*, an end-point PCR was used. The fragments containing this 6-bp Indel were amplified with the primer in Supplementary Table S1, then underwent electrophoresis in a 14% PAGE gel and resolved by silver staining.

### Overexpression of *E2F4* and *E2F6*

Overexpression of *E2F4* and *E2F6* were determined in IPEC-J2 cells. The complete CDS of porcine *E2F4* and *E2F6* was first amplified with the primer in Supplementary Table S1 and inserted between the *EcoR* I and *Xho* I or *Kpn* I sites of the pCMV-HA vector (Promega, Madison, WI, USA). The positive plasmids were confirmed by DNA sequencing and named pCMV-HA-E2F4 and pCMV-HA-E2F6, respectively. The expression of E2F4 and E2F6 were validated by Western blot analysis. In brief, IPEC-J2 cells transfected with E2F4 or E2F6 were harvested after lysing with RIPA buffer (SEVEN, Beijing, China), boiled with 5 × denaturing loading buffer and resolved in 12% sodium dodecyl sulfate-polyacrylamide gel electrophoresis (SDS-PAGE), then transferred to 0.22 μm of Immuno-Blot polyvinylidene fluoride (PVDF) membrane (Millipore, Billerica, MA, USA). Subsequently, the membrane was blocked with 5% BSA, washed with TBST and incubated in 1:1,000 dilution with a primary antibody for HA-tag (ABclonal, Wuhan, China) for 12 h at 4°C, and a secondary antibody for 2 h at room temperature. The membrane was washed with TBST and the bands were detected using the Super ECL kit (SEVEN, Beijing, China).

To validate the role of E2F4 and E2F6 on transcriptional activity of reporter gene carrying SNP1, IPEC-J2 cells were transfected with 0.25 μg of luciferase reporter vector pGL3-STAT3-P or pGL3-STAT3-SNP1, 0.25 μg of corresponding expression plasmid pCMV-HA-E2F4, pCMV-HA-E2F6 or pCMV-HA, and 0.005 μg of pRL-TK. Relative luciferase activity was calculated as described above.

### Electrophoretic mobility shift assay

The nuclear extracts of the IPEC-J2 cell transfected with pCMV-HA-E2F4 or pCMV-HA-E2F6 were prepared using the nuclear and cytoplasmic protein extraction kit (Eyotime, Shanghai, China) and quantified by the BCA method (Beyotime, Shanghai, China). Specific DNA probes for SNP1 A or G alleles were synthesized with or without a 5′-end biotin label (Tongyongshengwu, Anhui, China) in Supplementary Table S2. The EMSA assay was performed using a light chemiluminescent EMSA Kit (Beyotime, Shanghai, China) according to manufacturer instructions. Briefly, a reaction mixture containing 2 μg of nuclear protein, 2 μl of 5 × EMSA/Gel-Shift Binding Buffer, 1 μl of 500 μmol/μl biotin-labeled probes and 5 μl of distilled water (ddH_2_O) was incubated on normal atmospheric temperature for 20 min. For the competition assay, a 50-fold, 100-fold and 200-fold excess of unlabeled probes was added to the reaction mixture described above. Additionally, 1 μg of antibody for HA-tag (ABclonal, Wuhan, China) was added to the reaction mixture described above and incubated for 30 min at 4°C prior to incubation with the biotin-labeled probes. The DNA-protein complexes were then separated by electrophoresis on a 6.5% PAGE, transferred onto nylon membranes and cross-linked for 1 min with a UV cross-linker and the signal was detected in line with the manufacturer's instructions.

### Statistical analysis

The STAT3 mRNA expression level among the tissues or cells was analyzed using one-way ANOVA. The luciferase reporter activity between different reconstructions were evaluated using the *T*-test in GraphPad Prism 5 software (GraphPad, La Jolla, CA, USA). The observed heterozygosity (*Ho*), expected heterozygosity (*He*), effective allele numbers (*Ne*) and chi-square test for Hardy-Weinberg equilibrium of the genetic variation polymorphisms were calculated using Popgene software (version 1.32). The linkage disequilibrium (LD) of three functional loci were calculated by the online SHEsis software (http://analysis.bio-x.cn/myAnalysis.php). The haplotypes were build and the corresponding frequency were calculated using SAS version 8.0.

The effects of genotype or haplotype, breed and the sow on piglet diarrhea trait were analyzed using a general linear model (GLM) procedure of SAS version 8.0 according to the following statistical model:


$$\begin{array}{l} Y_{ijk}=\mu +G_{i}+B_{j}+S_{k}\;+e_{ijk} \end{array}$$

Where *Y*_*ijk*_ is the observed traits, μ is the population mean, *G*_*i*_ is the fixed effect of genotype or haplotype, *B*_*j*_ is the effect of breed, *S*_*k*_ is the maternal effect and *e*_*ijk*_ is the random residual.

The association analysis between genetic variation polymorphisms (or haplotype) and the piglets' phenotype traits in Min or Landrace population were assessed using a GLM procedure of SAS version 8.0 according to the following statistical model:


$$\begin{array}{l} Y_{ij}=\mu +G_{i}+S_{j}\;+e_{ij} \end{array}$$

Where *Y*_*ij*_ is the observed traits, μ is the population mean, *G*_*i*_ is the fixed effect of genotype or haplotype, *S*_*j*_is the maternal effect and *e*_*ij*_ is the random residual.

## Results

### Porcine *STAT3* mRNA expression in the tissues of piglets

**Figure 1:**
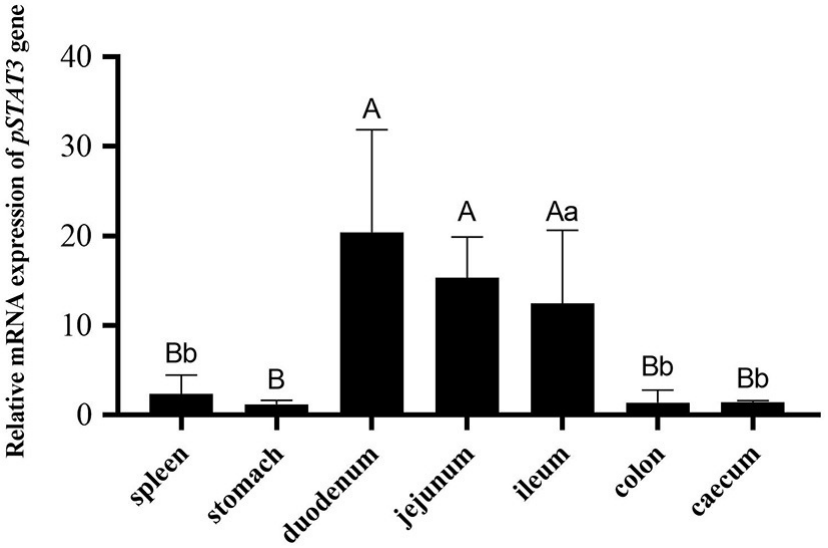
The mRNA expression of porcine STAT3 in the tissues of the 28-day SPF piglets (*n* = 3). The tissues expression data was analyzed by one-way ANOVA. Different capital letter indicated significant difference (*P* < 0.01); different lowercase letter indicated significant difference (*P* < 0.05). Data are presented as means ± SD.

RT-qPCR was used to confirm the expression of porcine *STAT3* in the gastrointestinal tract of SPF piglets. As show in Figure 1, porcine *STAT3* was expressed in all the examined tissues including spleen, stomach, duodenum, jejunum, ileum and cecum. Higher mRNA expression of *STAT3* existed in the small intestine including the duodenum, jejunum and ileum than in the tissues of the spleen, stomach and large intestine (Figure 1).

### Identification of genetic variations in the promoter region of *STAT3*

A 1906-bp fragment containing an 1134-bp 5′ flanking region, 410-bp exon 1 and 362-bp intron 1 of porcine *STAT3* was produced from 10 Min and 10 Landrace pigs by PCR. Nine SNPs and a 6-bp Indel were revealed through multiple sequence alignment (Supplementary Table S3). Among these variations, three SNPs were predicted to disrupt or create TFBS using both JASPAR and PROMO online bioinformatics tools in Table 1. For g.−870 G>A which was renamed SNP1, PROMO predicted that E2F-1 would bind to the DNA sequence of the G allele but not the A allele. Similarly, JASPAR predicted that E2F4/E2F6 would bind to the G allele but not the A allele. For g.−584 A>C which was renamed SNP2, both PROMO and JASPAR revealed that SP1 would bind to the C allele instead of the A allele. For the g.34 G>C, renamed SNP3, PROMO predicted that C/EBP beta would bind to the DNA sequence of the C allele but not the G allele. However, JASPAR predicted that RHOXF1 would bind to the SNP3 G allele but not the C allele. For the 6-bp Indel, PROMO predicted that myc-associated zinc finger protein (MAZ) would bind to the DNA sequence of the 6-bp insert but not the 6-bp deletion, while this variation does not disrupt or create a TFBS when using JASPAR.

**Table 1:** Putative transcriptional factor binding sites in the 5' non-coding region of porcine STAT3 gene.

**SNP^a^**	**Variation**	**Gain^b^**	**Loss^c^**	**Prediction database**
g.−870 G>A	G>A	E2F1	GR-alpha	PROMO
		E2F4	-	JASPAR
		E2F6	-	
g.−584 A>C	A>C	-	TFII-I	PROMO
		-	Sp1	
		-	SP1	JASPAR
		-	KLF5	
g.−543 T>G	T>G	-	GR-alpha	PROMO
g.−232 C>T	C>T	Pax-5	AP-2 alphaA	PROMO
		p53	GR-alpha	
		-	PXR-1:RXR-	
g.34 G>C	G>C	-	C/EBPbeta	PROMO
		RHOXF1	-	JASPAR
g.603 A>G	A>G	GR-beta	-	PROMO
		OTX1	-	JASPAR
6-bp Indel	6-bp Indel	MAZ	-	PROMO

a Based on position before exon 1;

b, c Generated after substitution of allele 1 (wild type) with allele 2 (mutant).

### Genotyping and population genetic analysis of three candidate functional genetic variations in porcine *STAT3*

**Figure 2:**
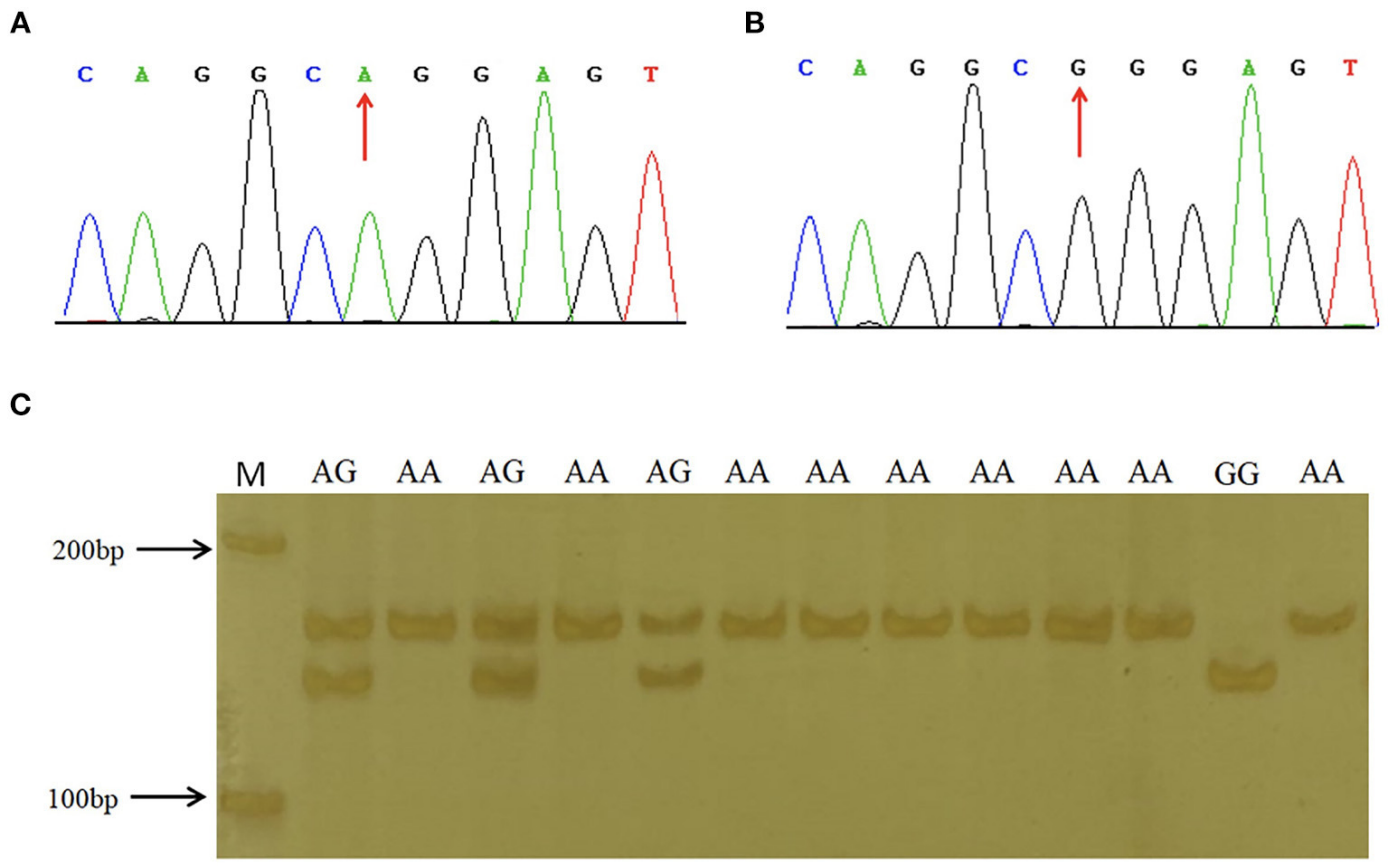
Sequence and genotyping results of SNP1 in porcine STAT3 promoter region. **(A,B)** show the sequencing results of SNP1 in the promoter region of porcine STAT3. **(C)** The enforced MspI PCR-RFLP assay for SNP1. Lane M molecular marker DL 2,000.

To explore the association between predicted functional variations and piglet phenotype, SNP1, SNP2 and the 6-bp Indel of porcine *STAT3* were genotyped in Min and Landrace populations. For SNP1, the PCR products from different animals were observed as AA with 158-bp, AG with 158-bp, 137-bp and 21-bp and GG with 137-bp and 21-bp, as seen in Figure 2. As shown in Table 2, the frequency of A allele was 0.44 in Min and 0.56 in the Landrace population. The *Ho* or *He* of SNP1 were 0.55 or 0.49 in Min pigs and 0.42 or 0.43 in Landrace pigs. The effective allele number (*Ne*) of Min and Landrace population was 1.97 and 1.77, respectively. The polymorphism information content (*PIC*) values were 0.37 and 0.34 in Min and Landrace pigs, respectively. The SNP2 was genotyped using the PCR-SSCP in Supplementary Figure S1. The A allele frequency of SNP2 was 0.52 in Min pigs, while this loci was fixed as SNP2-A in Landrace pigs as seen in Table 2. In Min pigs, the *Ho* and *He* of SNP2 were 0.47 and 0.50, respectively, the *Ne* was 2.00 and the *PIC* was 0.37. For the 6-bp Indel polymorphism, PCR products were observed as II with 124-bp, ID with 124-bp and 118-bp and DD with 118-bp after the PAGE in Supplementary Figure S2. The D allele or 6-bp deletion was absent in Min pigs, while its frequency was 0.24 in Landrace population (Table 2). In Landrace pigs, the *Ho* and *He* of this 6-bp Indel loci were 0.34 and 0.36, respectively. And the *Ne* and *PIC* values were 1.57 and 0.30, respectively. The chi-square test indicated that all these loci was in Hardy-Weinberg equilibrium.

**Table 2:** Genotype and allelic frequency of SNP1, SNP2 and 6-bp Indel of STAT3 gene in Min pig and Landrace populations.

**SNP**	**Breed**	**Genotype frequency**	**Allelic frequency**	***H_0_***	***He***	***Ne***	***PIC***	***χ^2^*** **(HWE)**
SNP1: g.−870 G>A	Min	AA 0.16 (37) AG 0.55 (125) GG 0.28 (64)	A 0.44 G 0.56	0.55	0.49	1.97	0.37	3.38
	Landrace	AA 0.47 (84) AG 0.42 (76) GG 0.11 (20)	A 0.68 G 0.32	0.42	0.44	1.78	0.34	0.20
SNP2: g.−584 A>C	Min	AA 0.29 (63) AC 0.47 (103) CC 0.24 (53)	A 0.52 C 0.48	0.47	0.50	2.00	0.37	0.72
	Landrace	AA 1 (180) AC 0 CC 0	A 1.00 C 0	-	-	-	-	-
6-bp Indel	Min	II 1 (226) ID 0 DD 0	I 1.00 D 0	-	-	-	-	-
	Landrace	II 0.5 (110) ID 0.34 (62) DD 0.07 (13)	I 0.76 D 0.24	0.34	0.36	1.57	0.30	1.06

*Ho* observed heterozygosity, *He* expected heterozygosity, *Ne* effective allele numbers, *PIC* polymorphism information content, χ*2 (HWE)* chi square test for Hardy–Weinberg equilibrium.

The LD among these three loci was revealed by the pairwise LD parameters (D' and *r*^2^). In Min population, the D' value between SNP1 and SNP2 was 0.83; the *r*^2^ was 0.50 (Supplementary Figures S3A,B). Four haplotypes were constructed and Hap4: GCI was the major haplotype with the frequency of 0.45 (Table 3). In Landrace population, the D' value between SNP1 and the 6-bp Indel was 0.94; the *r*^2^ was 0.13 (Supplementary Figures S3C,D). Four haplotypes were also found in Landrace piglets and the frequency of major haplotype Hap1: AAI was 0.44 (Table 3).

**Table 3:** Haplotype construction among three functional loci of porcine STAT3, haplotype frequency and association analysis in Min and Landrace population.

**Breed**	**Name**	**Hap1**	**Hap2**	**Hap3**	**Hap4**
Min	Haplotype	AAI	GAI	ACI	GCI
	Number	177	49	13	193
	Haplotype frequency (%)	0.41	0.11	0.03	0.45
	Diarrhea score	3.12 ± 0.19	2.56 ± 0.37	3.20 ± 0.71	2.63 ± 0.19
Landrace	Haplotypes	AAI	GAI	AAD	GAD
	Number	158	114	83	1
	Haplotype frequency (%)	0.44	0.32	0.23	-
	Diarrhea score	6.11 ± 0.42^a^	3.61 ± 0.50^Bb^	5.52 ± 0.57^a^	-

Different capital letters indicated significant difference (*P* < 0.01), Different lowercase letters indicated significant difference (*P* < 0.05).

### Association analysis of candidate functional genetic variations in porcine *STAT3* and phenotype traits

Statistical analysis showed that effects of SNP1 genotype, breed, and the maternity on phenotype trait were all significant (*P* < 0.01 or *P* < 0.05) (Table 4). However, it was not significant for the effect of SNP2 genotype, 6-bp Indel genotype and the haplotype (Table 4, Supplementary Table S4). The following association analysis indicated the diarrhea scores of the SNP1-GG individuals were lower than that of AA animals in both Min and Landrace populations (*P* < 0.01) as shown in Table 5. However, neither SNP2 nor the 6-bp Indel polymorphism affected piglet diarrhea score (Table 5). The phenotype difference among Min piglets with different haplotypes was not significant, however, Landrace individuals with Hap2:GAI had lower diarrhea score than Hap1:AAI (*P* < 0.05) or Hap3:AAD (*P* < 0.01) animals. Hap2:GAI might be resistant to Landrace piglet diarrhea, whereas Hap1:AAI and/or Hap3:AAD may be the susceptible haplotype. Collectively, the consistent statistic results in Min and Landrace breeds indicated piglets with SNP1-GG genotype were healthier than SNP1-AA carriers.

**Table 4:** Effects of genotype, breed and maternal effect of functional genetic variations in STAT3 gene on diarrhea score in Min pig and Landrace populations.

**Genetic variations**	**Genotype**	**Breed**	**Maternal effect**
SNP1: g.−870 G>A	3.28^*^	28.07^**^	5.47^**^
SNP2: g.−584 A>C	2.31	-	8.42^**^
6-bp Indel	1.85	-	8.46^**^

* Significant (P < 0.05),

** Significant (P < 0.01).

**Table 5:** Association between functional genetic variations in STAT3 gene and piglet diarrhea score in Min pig and Landrace populations.

**SNP**	**Breed**	**Genotype**	**Diarrhea score**
SNP1: g.−870 G>A	Min pig	AA	3.61 ± 0.41^A^
		AG	2.93 ± 0.23
		GG	2.25 ± 0.32^B^
	Landrace	AA	6.83 ± 0.56^A^
		AG	3.75 ± 0.59^B^
		GG	3.18 ± 1.16^B^
SNP2: g.−584 A>C	Min pig	AA	3.16 ± 0.33
		AC	2.77 ± 0.25
		CC	2.50 ± 0.36
6-bp Indel	Landrace	II	5.17 ± 0.52
		ID	4.99 ± 0.69
		DD	6.50 ± 1.51

Different capital letters indicated significant difference (P < 0.01).

### Effect of candidate genetic variations on *STAT3* transcriptional activity

**Figure 3:**
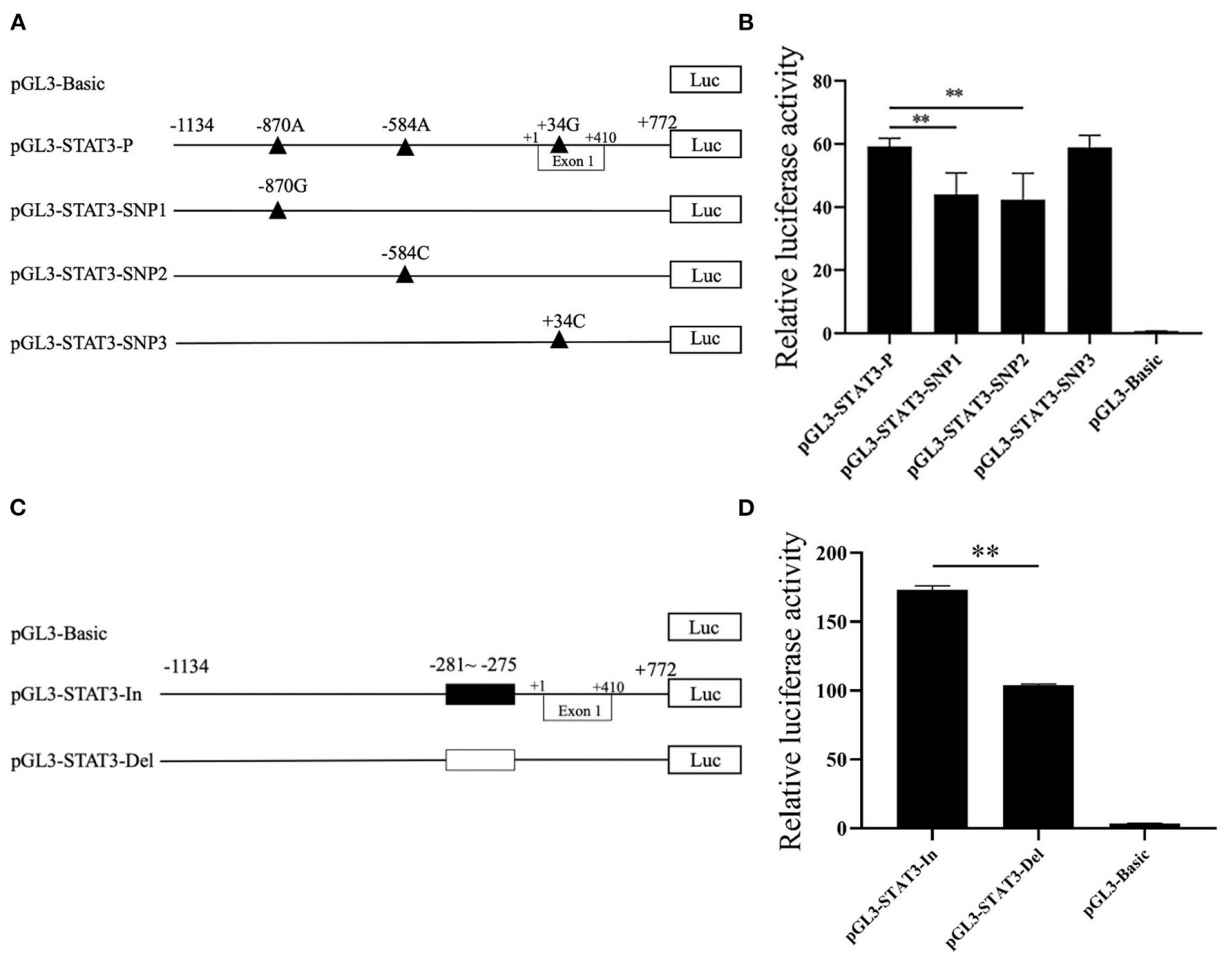
The effects of candidate genetic variations on porcine STAT3 transcriptional activity. **(A)** Schematic presentation of luciferase reporter construct for SNP1, SNP2, and SNP3. Genomic fragments carrying the SNPs were inserted the upstream of the SV40 promoter of the pGL3 vector. SNPs were named and numbered from the first nucleotide of the first Exon of porcine STAT3 (ENSSSCT00000018944.5) which was assumed as the putative transcriptional initial site and assigned as +1. **(B)** Luciferase assays of different SNPs luciferase reporter constructs in IPEC-J2 cells. **(C)** Schematic presentation of luciferase reporter constructs of the 6-bp Indel. **(D)** Luciferase assays of the 6-bp Indel in IPEC-J2 cells. Values are shown as the mean ± SD (*n* = 3). **P < 0.01.

To verify the effects of predicted functional genetic variations on *STAT3* transcriptional regulation, six luciferase reporter vectors including one control named pGL3-STAT3-P which containing SNP1 (−870A), SNP2 (−584A), SNP3 (+34G) and Indel (6-bp deletion), three single SNP reporter vectors including SNP1 (−870G), SNP2 (−584C) and SNP3 (+34C) and two 6-bp indel reporter vectors were constructed, as shown in Figures 3A,C. For these SNP reporter vectors, the luciferase activity of the control and three single SNP reporter vectors were compared. The luciferase activity of SNP1 G allele was lower than that of the A allele as in Figure 3B. Similarly, for SNP2, the C allele had lower luciferase activity than the A allele also in Figure 3B. However, the difference of luciferase activity between the C and G allele at SNP3 loci was not significant (Figure 3B). In addition, the statistical result indicated that the 6-bp insert owned higher luciferase activity when compared with the 6-bp deletion (Figure 3D). Interestingly, for the haplotype in Landrace population, the luciferase activity of major haplotype Hap1:AAI (pGL3-STAT3-In) was higher than Hap3:AAD (pGL3-STAT3-Del) as shown in Figure 3D.

### Effect of E2F4 or E2F6 on porcine *STAT3* transcriptional activity

**Figure 4:**
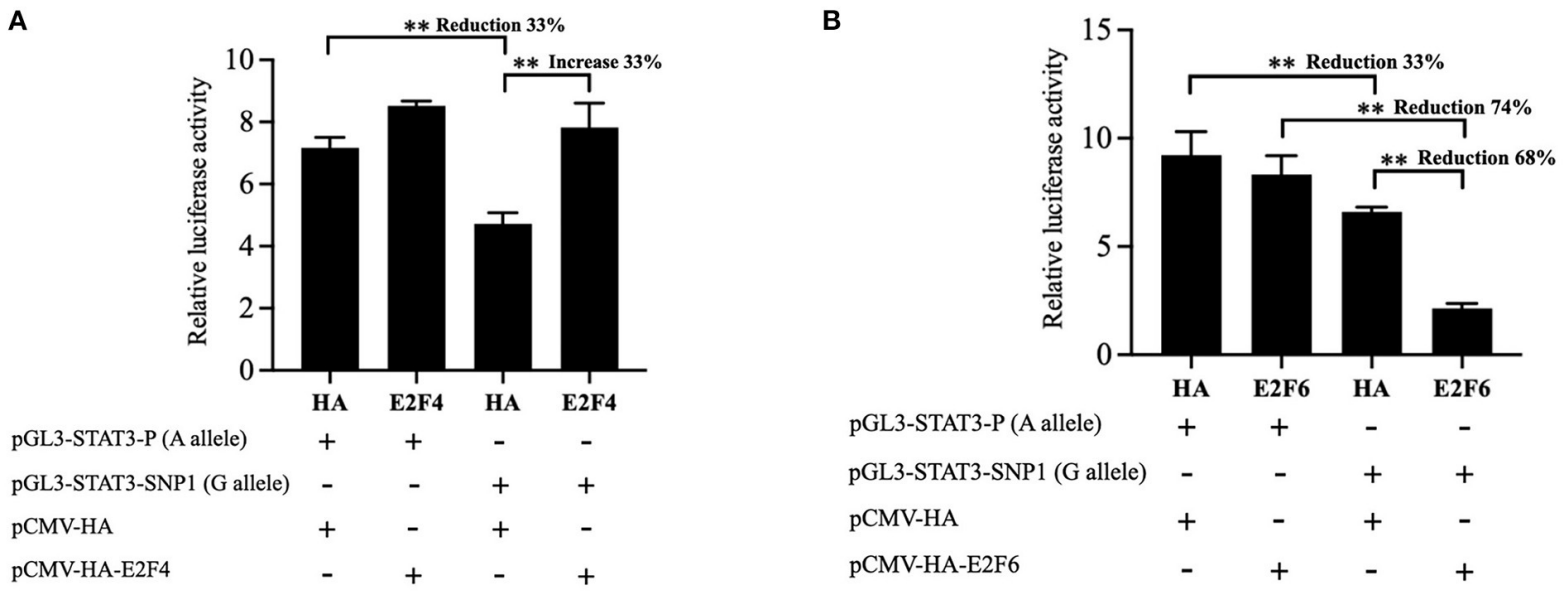
Effect of E2F4 and E2F6 on transcriptional activity of STAT3 gene carrying SNP1. **(A)** IPEC-J2 cells were transfected with the luciferase reporter construct containing A or G at STAT3 SNP1 and E2F4 expression vector. **(B)** IPEC-J2 cells were transfected with the luciferase reporter construct containing A or G at STAT3 SNP1 and E2F46 expression vector. Relative luciferase activity was given as firefly activity over Renilla activity. Values are shown as the mean ± SD (*n* = 3). **P < 0.01.

To further define the role of transcriptional factors on the transcriptional activity of the reporter gene carrying SNP1, the E2F4 or E2F6 expression vector were cotransfected into IPEC-J2 cells with the luciferase reporter construct pGL3-STAT3-P containing SNP1-A or pGL3-STAT3-SNP1 containing SNP1-G. Statistical results showed the overexpression of E2F4 significantly increased the luciferase activity of the SNP1-G by 34%, but not the A allele as in Figure 4A. E2F6 had no effect on the luciferase activity of pGL3-STAT3-P, while it significantly suppressed the G allele luciferase activity by 68%, as in Figure 4B.

### Binding of E2F4 and E2F6 to STAT3 *in vitro*

**Figure 5:**
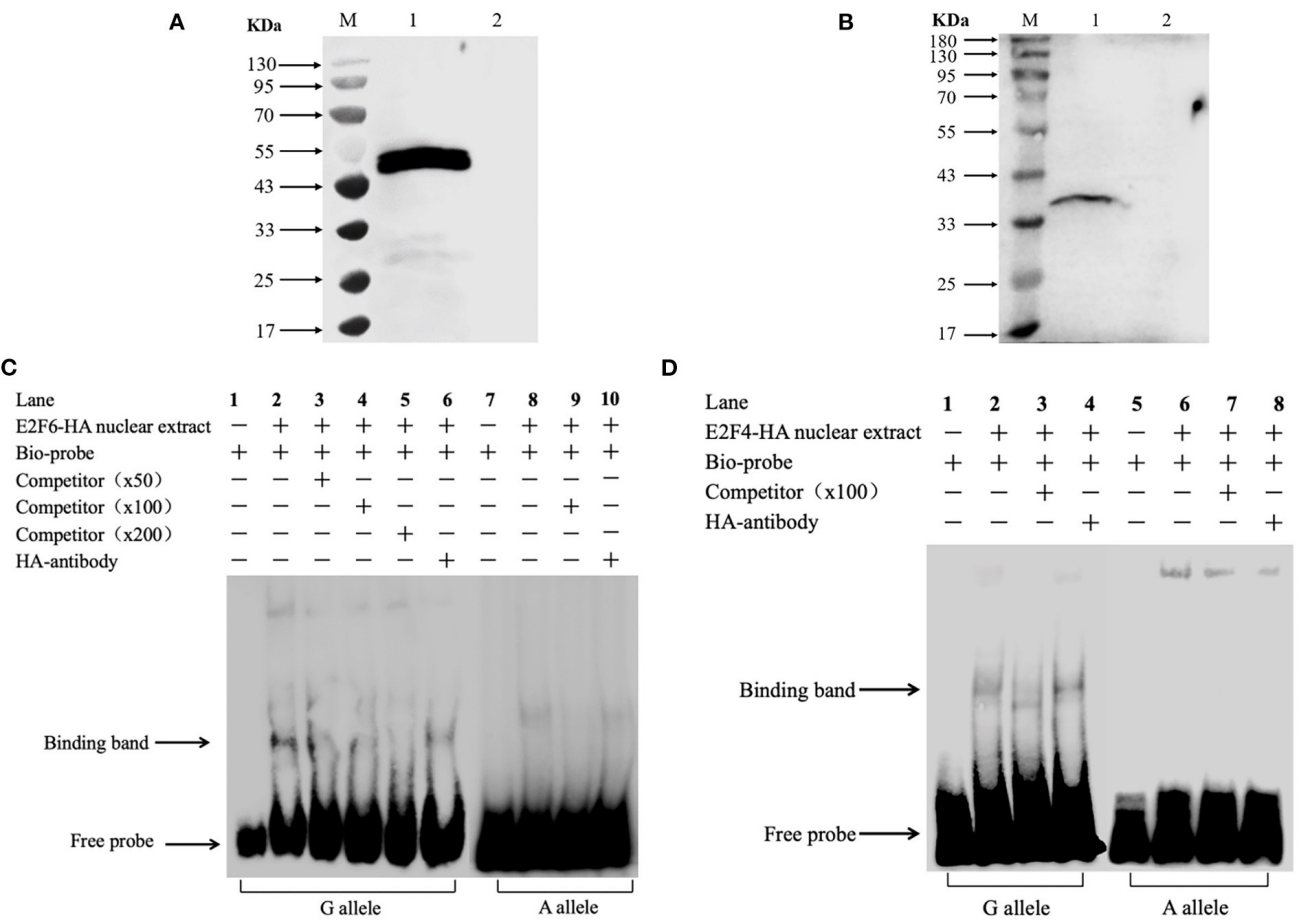
*In vitro* analysis of the interaction between transcription factors and STAT3 gene carrying SNP1. **(A)** Over expression of porcine E2F4 in IPEC-J2. Lane M: marker; Lane 1: IPEC-J2 transfected with pCMA-HA-E2F4; Lane 2: IPEC-J2 transfected with pCMA-HA. **(B)** Over expression of porcine E2F6 in IPEC-J2. Lane M: marker; Lane 1: IPEC-J2 transfected with pCMA-HA-E2F6; Lane 2: IPEC-J2 transfected with pCMA-HA. **(C)** The binding of E2F4 on the promoter of STAT3 gene. **(D)** The binding of E2F6 on the promoter of STAT3 gene. Arrows represent the free probe and the specific band for the GG probe. Lane 1: Free probe containing the G allele is observed. Lane 2: Binding of nuclear proteins is observed with the probe containing the G allele. Lane 3: Binding was competed by unlabeled probe.

Integrating the above association analysis and the bioinformatics predictions, an *in vitro* EMSA was performed to verify the effect of SNP1 on the binding of E2F4 or E2F6 to the cis-acting element of porcine STAT3. The western blot results showed that E2F4-HA or E2F6-HA existed at elevated levels in nuclear extracts derived from IPEC-J2 cells transfected with the respective expression vector in Figures 5A,B. The results of the EMSA showed that the probe harboring the G allele of SNP1 formed a DNA-protein complex with nuclear extracts rich in E2F4-HA, but no binding was detected on the A allele in Figure 5C. Similarly, the probe harboring SNP1-G allele, instead of SNP1-A allele, formed a DNA-protein complex with nuclear extracts rich in E2F6-HA in Figure 5D.

## Discussion

The small intestine plays important roles in digestion, absorption and immune function, contributing to the maintenance of individual health. Existing evidence revealed the replication of major porcine diarrhea-associated viruses, including porcine epidemic diarrhea virus (PEDV), transmissible gastroenteritis virus (TGV), porcine delta coronavirus (PoDV) and swine acute diarrhea syndrome coronavirus (SADS-CoV), was limited to intestinal villus epithelial cells (19, 30). Early weaning stress in piglets may also cause intestinal dysfunction, which results in the activation of gut inflammation, further thus increasing the incidence of diarrhea (31, 32). The mRNA expression of porcine *STAT3* was altered significantly in IPEC-J2 after infection by PEDV or in the jejunum of piglets during weaning (21, 22), but its expression in the gastrointestinal tract of sucking piglets was unknown. This study found *STAT3* was expressed in all selected tissues with higher levels in the ileum, jejunum and duodenum and this higher expression suggests a significant role in the intestinal health of sucking piglets.

In the past decade, accumulated evidence have shown that most of the disease or complex trait associated variants discovered in genome-wide association studies (GWAS) were in non-coding sequences and that several variants were involved in the transcriptional regulatory of a candidate gene (33–35). Although the GWAS method has been applied to the field of animal breeding and genetics, variants that are statistically associated with piglet diarrhea have not been reported. And in this study, candidate gene strategy was employed to explore causative variants. Putative functional genetic variations in porcine *STAT3* promoter region were first screened by *in silico* tools and the subsequent luciferase reporter assay verified that SNP1 and SNP2, which were predicted to alter transcriptional factor binding sites by both JASPAR and PROMO tools showed diverse transcriptional activity. Considering the key role of CEBPB in the immunity (36, 37) and the novel role of porcine CEBPB in the transcription regulation of porcine *CXCL14* which contributes to PRRS resistance (6), SNP3 was selected to process the functional verification. However, SNP3 could not affect the luciferase activity, which indicated that CEBPB might not be a high confidence transcription factor or this CEBPB polymorphism cannot alter the transcription activity of porcine *STAT3*. Interestingly, a repressive effect on luciferase activity of the 6-bp deletion which losing a putative MAZ binding site was found. Overall, SNP-1, SNP-2 and the 6-bp Indel which affected *STAT3* transcriptional efficiency *in vitro*, were potentially functional mutations.

Association analysis suggested that SNP1 rather than SNP2 or the 6-bp Indel was associated with piglet diarrhea and the frequency of the resistant allele SNP1-G present in the Min pig was higher than in the Landrace. It has been observed that Min and Landrace breeds vary in their capacity to resist diarrhea (25), so the findings of this study were consistent with the characteristics of these two breeds and association analysis provided evidence supporting a role for SNP1 in piglet diarrhea. However, more studies in large populations with different genetic background are necessary before this marker could be applied for selection breeding.

According to JASPAR, E2F4 and E2F6 can bind the SNP1-G allele but not the A allele and the EMSA results verified this prediction. The overexpression of E2F6 suppressed the luciferase activity of SNP1 G alleles by 68%, while E2F4 increased G alleles by 34%. Both E2F4 and E2F6 belong to the ubiquitous E2F family of transcription factors which control cell proliferation and terminal fate (38–40). E2F4 was initially categorized as the major repressor of cell cycle progressions, however, recent research showed that E2F4 acted in part as a transcriptional activator that promoted the expression of cell cycle genes in mouse embryonic stem cells (41), functioned as a positive regulator of milk biosynthesis and proliferation of bovine mammary epithelial cells (42) and it could positively regulate hepatitis B virus (HBV)transcription by binding the core promoter of HBV covalently closed circular DNA (43). In this study, the positive regulation of E2F4 on G allele supported the point that E2F4 regulated diverse gene expression programs in cell fate decisions (44).

Compared to E2F4, E2F6 lacks the C-terminal domain which contains a transactivation domain and the binding domain for pRB family members (39), and it can behave as a dominant negative inhibitor of the other E2F family members (45). The repression of E2F6 on the SNP1 G allele of *STAT3* was consistently found in this study. Considering the positive role of *STAT3* on PEDV replication (20) and the low diarrhea score of SNP1-GG piglets found in this study, it is hypothesized that E2F6 binds as a repressor to the promoter containing the SNP1 G allele, downregulates the transcription of *STAT3* and then decreases the replication of PEDV. Human papillomavirus type 16 E7 (HPV E7) and adenoviral E1A protein can interact with E2F6 to inactivate the transcriptional repression activity of E2F6 and subvert the critical cellular defense (46), but it is unclear whether E2F6 plays a role in the infection of PEDV.

In this study, SNP1 was identified as a functional variant, however, it remains unclear whether this SNP alters the transcription of *STAT3* through other pathways, such as DNA methylation or histone modification. Existing studies indicate the promoter methylation regulates candidate gene transcription and the binding of transcription factors at CpG islands contributes to protect DNA methylation (47–49). The most recent studies found E2F6 binds preferentially to CpG islands genome-wide in mouse embryos (50, 51). This study found SNP1 located in the predicted CpG islands of *STAT3* promoter. Ongoing work will focus on clarifying whether this E2F6 binding polymorphism confers methylation protection and contributes to the regulation of the expression of *STAT3*.

## Conclusion

In conclusion, our study demonstrated g.−870 G>A in porcine STAT3 promoter may influence piglet diarrhea through an association study and g.−870 G>A was characterized as an functional mutation that has an allele-specific effect on STAT3 transcription through varying affinity for E2F4 and E2F6. These findings suggest that g.−870 G>A might influence susceptibility to piglet diarrhea partially by modulating STAT3 transcription.

## Data availability statement

The original contributions presented in the study are included in the article/Supplementary material, further inquiries can be directed to the corresponding author.

## Ethics statement

The animal study was reviewed and approved by Laboratory Animal Management Committee of Northeast Agricultural University.

## Author contributions

BN and DG designed and supervised the experiments. ZC, DY, LL, and MK conducted the experiments. ZC and DY analyzed the data. YS and DG provided the SPF animals. XW, SD, and JC provided the Min pigs and Landrace animals. BN and ZC wrote the manuscript. DG and XY revised the manuscript. All authors contributed to the article and approved the submitted version.

## Funding

This work was supported by Foundation for Key Teacher of Northeast Agriculture University (19XG11) and the Natural Science Foundation of Heilongjiang Province (LH2020C015).

## Conflict of interest

The authors declare that the research was conducted in the absence of any commercial or financial relationships that could be construed as a potential conflict of interest.

## Publisher's note

All claims expressed in this article are solely those of the authors and do not necessarily represent those of their affiliated organizations, or those of the publisher, the editors and the reviewers. Any product that may be evaluated in this article, or claim that may be made by its manufacturer, is not guaranteed or endorsed by the publisher.
