# Supplementary material for: A functional mutation associated with piglet diarrhea partially by regulating the transcription of porcine STAT3
Source: Front Vet Sci. 2022 Nov 4;9:1034187. doi: 10.3389/fvets.2022.1034187 (PMC9672087; doi:10.3389/fvets.2022.1034187)
Supplement: Supplementary file 1 [file Data_Sheet_1.docx]

**Table S1.** Primers used for RT-qPCR, SNPs identification and plasmids construction

| **Prime** | **Primer sequence (5’-3’)** | **Annealing**  **temp(℃)** | **Product**  **size(bp)** | **Binding**  **region** |
| --- | --- | --- | --- | --- |
| STAT3 | F:ATCTCCAGGATGACTTTGAT | 56 | 203 | Exon 6 |
|  | R:AGTTTTCTGCACATACTCCA |  |  | Exon 8 |
| GAPDH | F:CCCCAACGTGTCGGTTGT | 55 | 83 | Exon 8 |
|  | R:CCTGCTTCACCACCTTCTTGA |  |  | Exon 9 |
| STAT3-P | F:AGGCACGCAATTTCAAGTA | 58 | 1906 | -1134 |
|  | R:ACAATCCAGTAAACGCAAGG |  |  | Intron 1 |
| SNP1-Msp I | F:GAAACTGAGGTTCAAAGCAGCC | 58 | 158 | -892 |
|  | R:CGTGGGGGTGAGTTAGGAGC |  |  | -735 |
| SNP2-SSCP | F:CGCATCAGGGGCATTTAAAGT | 56 | 143 | -691 |
|  | R:TCCGCAACAGTAAGGGAAGAGC |  |  | -549 |
| Indel-PCR | F:GGCTGGAGGGGCTGTAAT | 54 | 124 | -330 |
|  | R:GGGGTGCTCGTCAAGGAT |  |  | -207 |
| pGL3-P | F:GGAGGTACCAGGCACGCAATTTCAAGTA | 58 | 1906 | -1134 |
|  | R:GCGACGCGTACAATCCAGTAAACGCAAGG |  |  | Intron 1 |
| pGL3- SNP1 | F:GTTCAAAGCAGGCGGGAGTCACC | - | - | - |
|  | R:GGTGACTCCCGCCTGCTTTGAAC |  |  |  |
| pGL3- SNP2 | F:TCCGGGTCTCTCCGCCCAAGG | - | - | - |
|  | R:CCTTGGGCGGAGAGACCCGGAG |  |  |  |
| pGL3- SNP3 | F:AGTAGTGGACACAAGCTTCAGTAGG | - | - | - |
|  | R:CCTACTGAAGCTTGTGTCCACTACT |  |  |  |
| pCMV-E2F4 | F:TTCGAATTCCCATGGCGGAGGCCGGGCCACA | 58 | 1221 | - |
|  | R:ATTCTCGAGTCAGAGGTTGAGAACAGGCACATC |  |  |  |
| pCMV-E2F6 | F:TTCGAATTCGAATGAGTCAGCAGCGGCCG | 58 | 849 | - |
|  | R:CGGGGTACCTCAGTTGCTCACTTCAAGCAG |  |  |  |

**Table S2.** Probes used for EMSA

| **Probe** | **Prime** | **Primer sequence (5’-3’)** |
| --- | --- | --- |
| Biotinylated probe | SNP1-G-bio | F:GTTCAAAGCAGGCGGGAGTCACCCA-bio |
|  |  | R:TGGGTGACTCCCGCCTGCTTTGAAC-bio |
|  | SNP1-A-bio | F:GTTCAAAGCAGGCAGGAGTCACCCA-bio |
|  |  | R:TGGGTGACTCCTGCCTGCTTTGAAC-bio |
| Cold competitive probe | SNP1-G | F:GTTCAAAGCAGGCGGGAGTCACCCA |
|  |  | R:TGGGTGACTCCCGCCTGCTTTGAAC |
|  | SNP1-A | F:GTTCAAAGCAGGCAGGAGTCACCCA |
|  |  | R:TGGGTGACTCCTGCCTGCTTTGAAC |

| **Table S3.** Identification of genetic variations in the 5’ non-coding region of porcine *STAT3* from Min pig and Landrace | **g.+603 A>G (rs338303469)** | A/G | 603 | A | G | A | G | G | G | G | G | A | A | G | G | G | G | A | G | G | G | G | A | Note: SNPs are named and numbered from the first nucleotide of the first Exon of porcine STAT3 (ENSSSCT00000018944.5) which is assumed as the putative transcriptional initial site and assigned as +1. |
| --- | --- | --- | --- | --- | --- | --- | --- | --- | --- | --- | --- | --- | --- | --- | --- | --- | --- | --- | --- | --- | --- | --- | --- | --- |
|  | **g.+63 A>G (rs81432389)** | A/G | 63 | G | G | G | G | G | G | G | G | A | G | G | G | G | G | A | G | G | G | G | G |  |
|  | **g.+34 G>C (rs324619040)** | G/C | 34 | C | C | C | G | G | G | G | G | G | C | G | G | G | G | G | G | G | G | G | C |  |
|  | **g.-232 C>T (rs322400358)** | C/T | -232 | T | T | T | C | C | C | C | C | C | T | C | C | C | C | C | C | C | C | C | T |  |
|  | **6-bp Indel** | CCGGGA >- | −281~-275 | CCGGGA | CCGGGA | CCGGGA | CCGGGA | CCGGGA | CCGGGA | CCGGGA | CCGGGA | CCGGGA | CCGGGA | ------ | ------ | ------ | CCGGGA | CCGGGA | ------ | CCGGGA | CCGGGA | CCGGGA | CCGGGA |  |
|  | **g.-402 A>G (rs345021211)** | A/G | -402 | G | G | G | G | G | G | G | G | A | G | G | G | G | G | A | G | G | G | G | G |  |
|  | **g.-543 T>G (rs323551092)** | T/G | -543 | G | G | G | T | T | T | T | T | T | G | T | T | T | T | T | T | T | T | T | G |  |
|  | **g.-584 A>C (rs333704989)** | A/C | −584 | C | C | C | A | A | A | A | A | A | C | A | A | A | A | A | A | A | A | A | C |  |
|  | **g.-812 T>C (rs345809666)** | T/C | -812 | C | T | T | T | T | T | T | T | T | T | T | T | T | T | T | T | T | T | T | C |  |
|  | **g.-870 G>A (rs327720240)** | G/A | −870 | G | G | G | A | A | A | A | A | G | G | A | A | A | A | G | A | A | A | A | G |  |
|  | **SNPs ID** | Mutation | Genome localization | Min-1-healthy | Min-2-healthy | Min-3-healthy | Min-4-healthy | Min-5-healthy | Min-1-diarrhea | Min-2-diarrhea | Min-3-diarrhea | Min-4-diarrhea | Min-5-diarrhea | Landrace-1-healthy | Landrace-2-healthy | Landrace-3-healthy | Landrace-4-healthy | Landrace-5-healthy | Landrace-1-diarrhea | Landrace-2-diarrhea | Landrace-3-diarrhea | Landrace-4-diarrhea | Landrace-5-diarrhea |  |

**Table S4.** Effects of procine STAT3 haplotype and maternal effect on diarrhea score in Min pig and Landrace populations

| Breed | Haplotype | Maternal effect |
| --- | --- | --- |
| Min pig | 1.36 | 17.73^**^ |
| Landrace | 0.92 | 20.00^**^ |

**Significant (*P* <0.01)


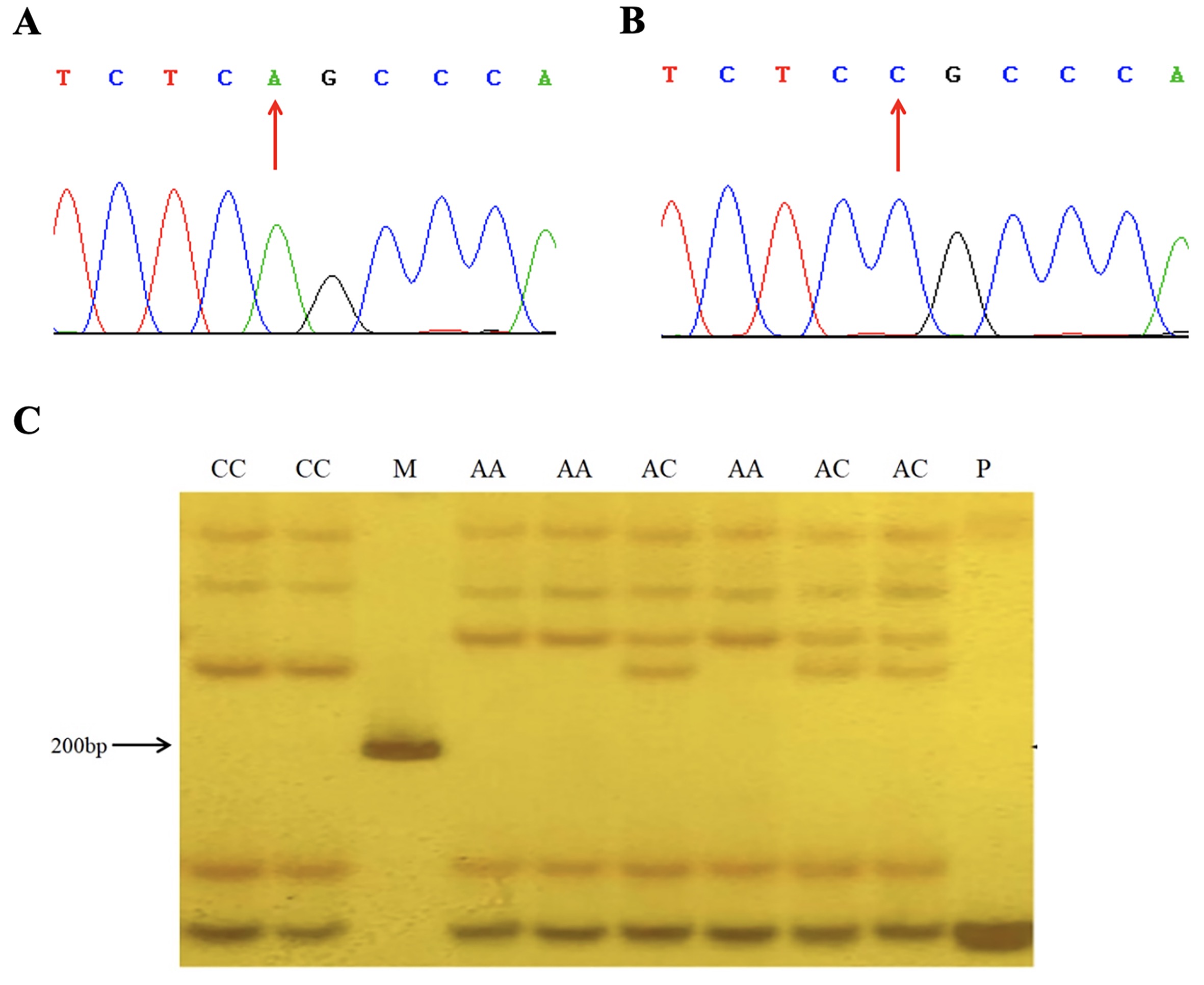


**Figure S1.** Sequence and genotyping results of SNP2 in porcine STAT3 promoter region. (A) and (B) show the sequencing results of SNP2 in the promoter region of porcine STAT3. (C) The PCR-SSCP for SNP2. Lane M: molecular marker DL2 000. Lane P: PCR product.


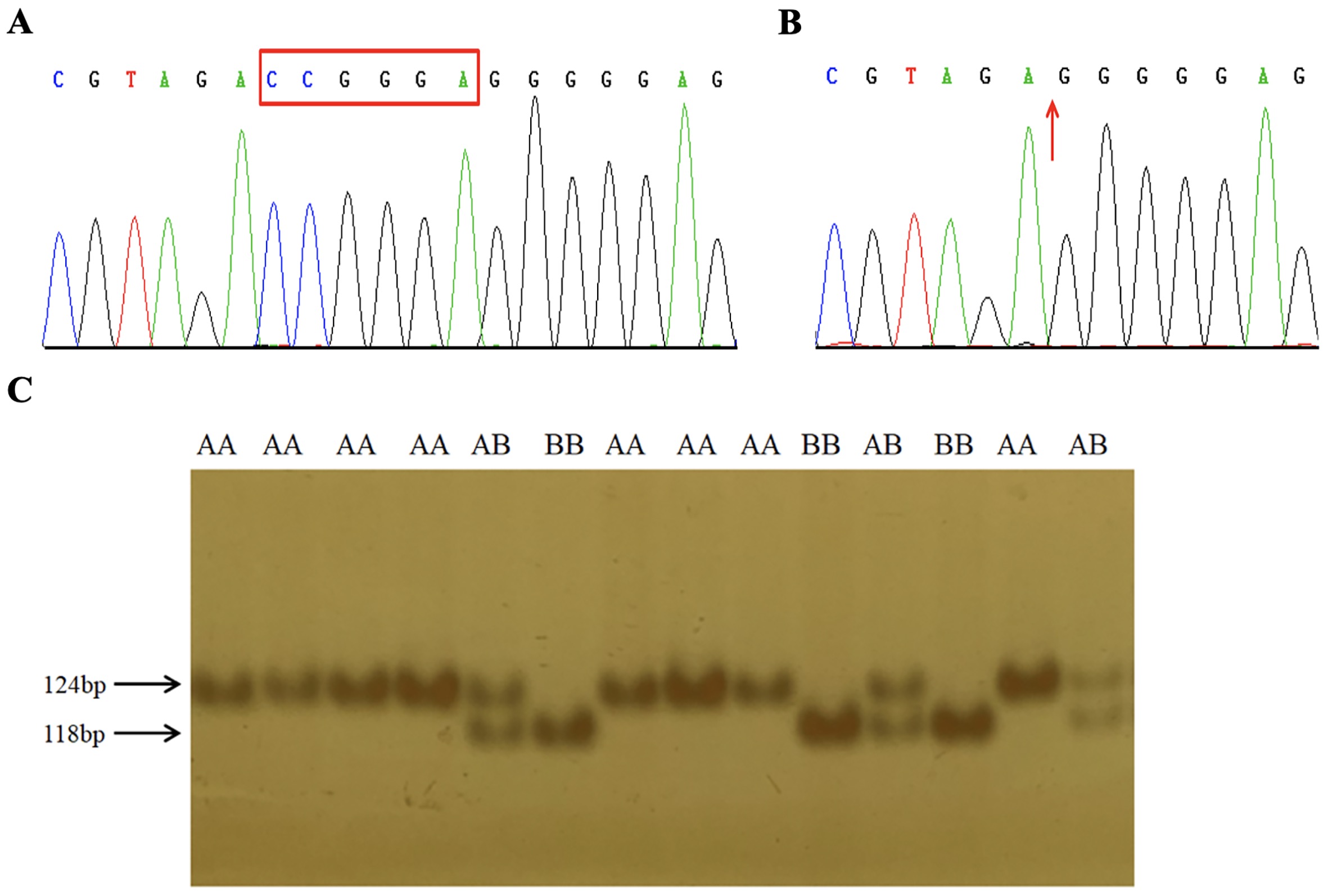


**Figure S2.** Sequence and genotyping results of the 6-bp indel in porcine STAT3 promoter region. (A) and (B) show the sequencing results of the 6-bp indel in the promoter region of porcine STAT3. (C) The end-point PCR assay for the 6-bp indel.


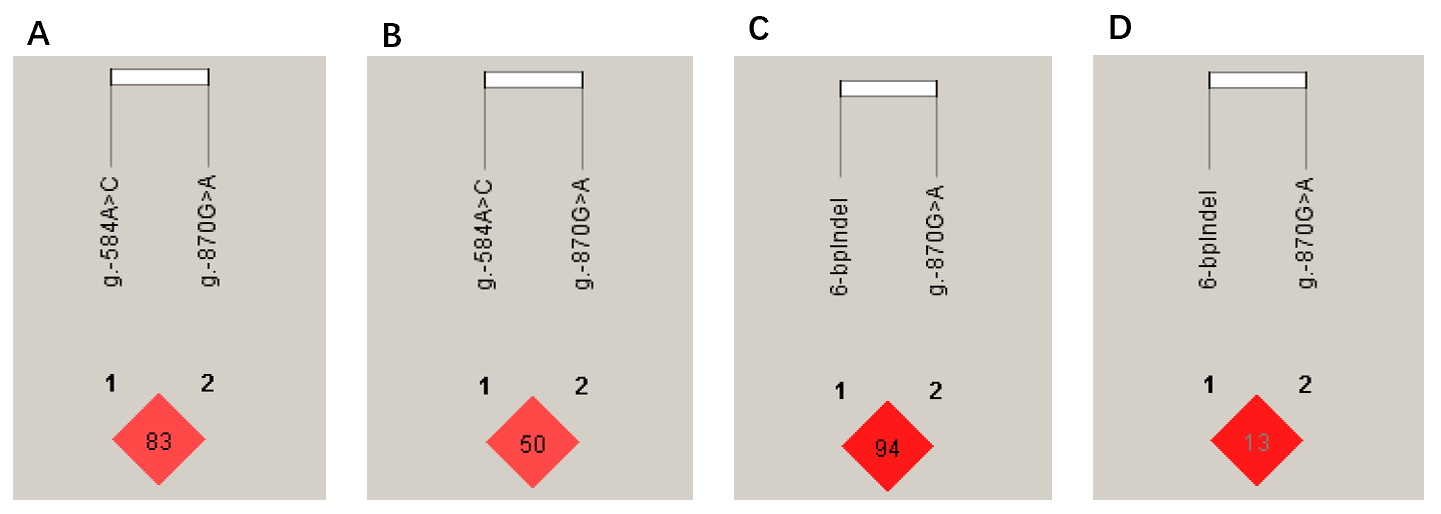


**Figure S3.** The linkage disequilibrium parameter (D’ and r^2^) among three functional loci of porcine STAT3 in Min and Landrace population, respectively. (A) The D’ value between SNP1 and SNP2 in Min pig. (B) r^2^ value between SNP1 and SNP2 in Min pig. (C) The D’ value between SNP1 and the 6-bp Indel in Landrace. (D) r^2^ value between SNP1 and the 6-bp Indel in Landrace.
